# Perioperative management of scoliosis surgery in child with epilepsy on the ketogenic diet: Case report

**DOI:** 10.1097/MD.0000000000043125

**Published:** 2025-06-27

**Authors:** Fangjun Yang, Dong Hou, Jiantao Wen

**Affiliations:** a Department of Orthopedic, Gansu Provincial Hospital of Traditional Chinese Medicine, Lanzhou, China; b School of Integrated Chinese and Western Medicine, Gansu University of Chinese Medicine, Lanzhou, China; c Department of Spinal Orthopedics, Gansu Provincial Hospital of Traditional Chinese Medicine, Lanzhou, China.

**Keywords:** ketogenic diet, perioperative period, case report, scoliosis

## Abstract

**Rationale:**

The ketogenic diet (KD) is a dietary regimen characterized by high-fat, moderate-protein, and extremely low-carbohydrate intake. While clinically established as an effective therapy for epilepsy and various metabolic and neurological disorders, its application presents unique challenges in surgical patients requiring perioperative maintenance of ketosis.

**Patient concerns:**

This report details the multidisciplinary management of a 12-year-old girl who was treated with KD for epilepsy for 10 years, who underwent posterior spinal fusion for scoliosis. At the same time, the need for perioperative care for KD-dependent pediatric patients undergoing complex procedures was also highlighted.

**Diagnoses:**

Imaging features showed scoliosis. The previous medical history indicates 10 years of KD treatment for epilepsy, intellectual disability for 10 years, and autism for 5 years.

**Interventions:**

The patient underwent spinal orthopedic surgery after real-time metabolic monitoring (blood glucose/ketone body detection) combined with a tailor-made anesthesia regimen.

**Outcomes:**

The scoliosis surgery was carried out smoothly, no abnormal conditions occurred during the perioperative period.

**Lessons:**

Maintenance of ketosis is critical throughout the perioperative period to prevent complications including hypoglycemia, seizures, surgical failure, or even mortality in severe cases. At the same time, the use of carbohydrate-free lipid infusion and propofofo-based anesthesia during fasting avoided inadvertent carbohydrate exposure, demonstrating adaptability to perioperative period.

## 1. Introduction

Ketogenic diet (KD) is a dietary method with high fat, low carbohydrate and moderate protein.^[1]^ The concept was first proposed by Wilder in 1921 to simulate starvation to control seizures. Ketone bodies produced by fatty acid metabolism as the main energy source can have anticonvulsant effects on the brain. Although its specific anticonvulsant mechanism is not clear, it has been widely used in a variety of metabolic disorders (such as obesity, diabetes mellitus), nervous system disorders (such as neuritis), and psychiatric disorders (such as depression, mood disorders, and anxiety).^[2–5]^ With the wide application population, surgeons are increasingly encountering a variety of KD patients requiring surgical treatment. However, so far, there are few case reports on the perioperative management of KD patients at home and abroad. We used remimazolam, a novel ultra-short-acting benzodiazepine, for induction of anesthesia to reduce the risk of seizures. The drug alleviates anesthesiologists’ concern about inducing or worsening seizures. Although, in surgery requiring intraoperative neurophysiological monitoring (IONM), propofol is still used as total intravenous anesthesia, gold standard,^[6]^ but the high carbohydrate load of propofol may disrupt ketosis and lower seizure threshold. Therefore, remimazolam is preferentially used to maintain strict carbohydrate restriction while ensuring hemodynamic stability and rapid recovery, which is critical for the accuracy of neuromonitoring.

This article reports the diagnosis and treatment of a case of scoliosis complicated with KD in the perioperative management, aiming to provide reference for the perioperative management of KD patients.

## 2. Case presentation

**Figure 1.**
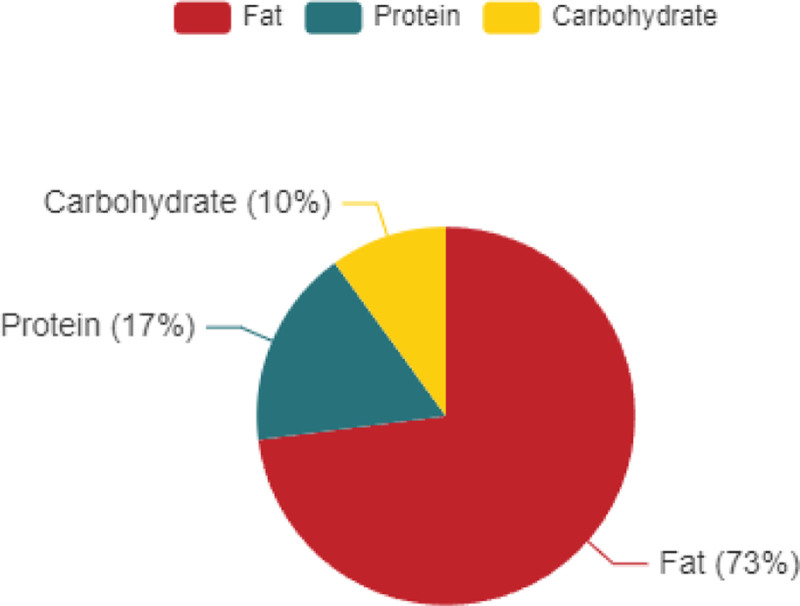
Macronutrient composition of the ketogenic diet therapy (KD).

We present a 12-year-old girl who was noted by her mother to have shoulders, hips uneven and prominent shoulder blade 2 years ago. She had a history of epilepsy for 12 years, intellectual disability for 10 years, and autism for 5 years. KD treatment of epilepsy for 10 years, daily intake of 220 g, total energy of 1430 kcal, the specific proportion is shown in Figure 1.

**Figure 2.**
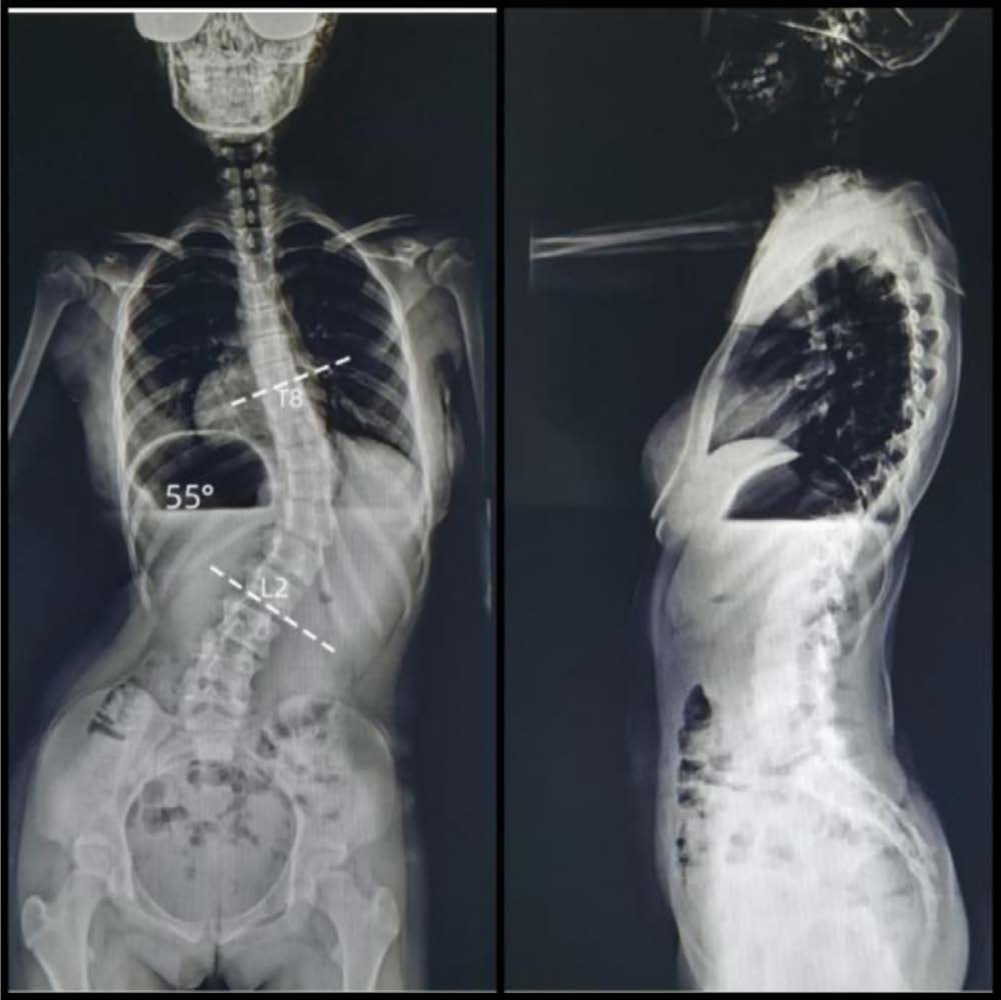
Preoperative full-length digital radiography (DR) of the spine, anteroposterior view demonstrating a right thoracic scoliosis with a Cobb angle of 55° (measured between T8 and L2 vertebrae). The image reveals significant spinal curvature consistent with moderate-to-severe scoliosis (Cobb angle > 50°), vertebral rotation, and compensatory lumbar counter-curvature. Imaging was performed in a standardized standing position. No instrumentation or prior surgical intervention is noted.

Upon admission to the hospital, laboratory tests were normal. The full-length spine digital radiography showed that the thoracolumbar T12 vertebral body showed a right curve (Fig. 2) with Cobb = 55°, which was considered as severe scoliosis and required surgical treatment.

During the preoperative evaluation, multidisciplinary consultation including pediatrics, anesthesiology, neurology, nursing, and nutrition departments was invited to formulate a personalized perioperative plan to ensure the safety of the children. KD was strictly followed before surgery. The fasting blood ketone level was 0.1 mmol/L before going to bed 1 day before surgery and 0.7 mmol/L at 6 am on the day of surgery, respectively (the normal value is 0.03–0.30 mmol/L). Oral KD was taken at 6 o ‘clock on the day of operation, which was better than that of 1/3 day before operation and 12 o ‘clock in the operation room, which met the requirements of preoperative fasting for elective surgery. The patients were trained to move their toes according to the instructions before operation, so as to facilitate intraoperative wake-up observation and ensure the safety of surgery.

After adequate preoxygenation, anesthesia was induced with remifentanil 20 μg (for analgesia), midazolam 1 mg, propofol 20 mg (for sedation), and cisatracurium 10 mg (for muscle relaxation). Anesthesia was maintained with remifentanil (analgesia) and remimazolam besylate (sedation). Frequency 16 breaths/min, inspiratory/expiratory ratio 1:2). Tracheal intubation (6. 5#, 21 cm) was performed under the guidance of video laryngoscope, and the internal jugular vein puncture and catheterization were performed under the guidance of ultrasound, and the right radial artery puncture and catheterization were performed. The motor evoked potential was connected at the beginning of the above operation to facilitate intraoperative neurophysiological monitoring.

The scoliosis surgery was carried out smoothly, and there was no abnormality in intraoperative neurophysiological monitoring. The intraoperative blood loss was 400 mL. The patient recovered spontaneous breathing after surgery (at 20 o ‘clock). After sputum suction and extubation, the patient was irritable, crying, and did not cooperate well. A 3# laryngeal mask was inserted after sedation. Two hours after admission to ICU, under the comfort of the child’s mother, the child’s mood gradually stabilized, the laryngoscope was successfully removed, and 2/3 of the remaining nutrition was injected intravenously. During the operation, blood glucose and blood ketone monitoring were strengthened, and fluid infusion was timely and effectively adjusted. Blood glucose and blood ketone were controlled in the normal range (Table 1).

**Table 1:** Intraoperative monitoring indexes (mmol/L).

Time	Blood glucose	Blood ketone
13:14	5	0.4
15:40	4.6	0.7
17:15	4.2	0.7
20:20	5.7	0.6

On the second postoperative day, the child did not defecate or exhaust, and was given 20 mL rectal enema, continuous fasting, adequate water, and cefazolin sodium to prevent infection. Intravenous nutrition was administered according to the KD. The specific dose was as follows: long chain fat emulsion injection 500 mL; 10% glucose injection 500 mL; compound amino acid injection 250 mL; sodium chloride 250 mL + vitamin C 0.5 g, the rate was about 50 mL/h, the temperature was 37°C, and the child tolerated it well.

**Figure 3.**
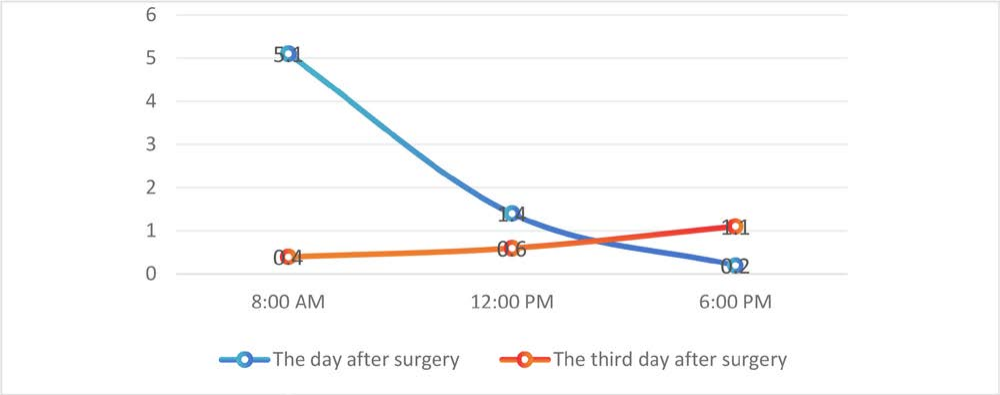
Diurnal variations in blood ketone concentrations.

**Figure 4.**
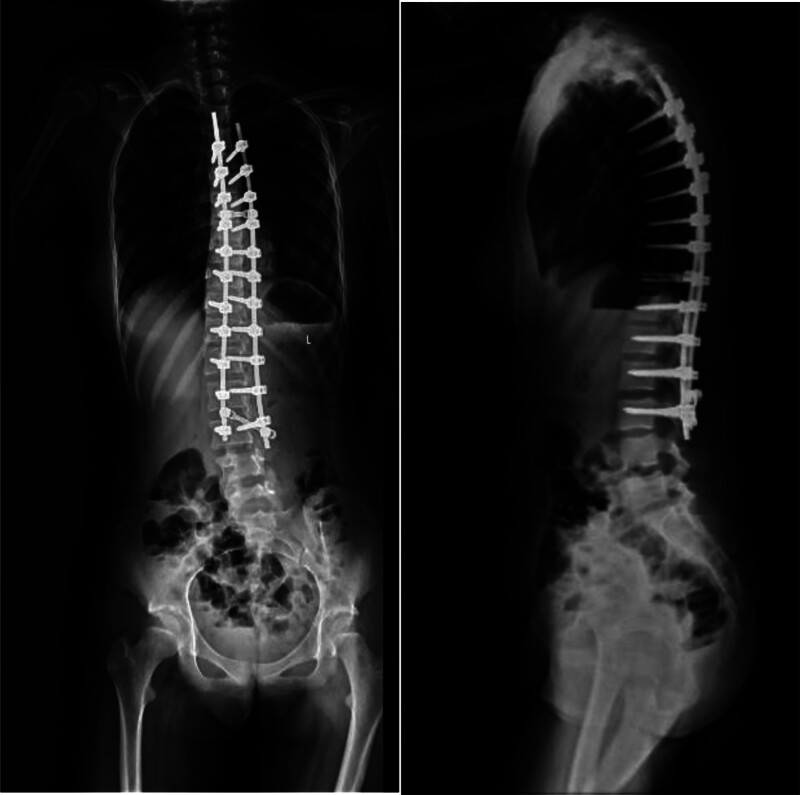
Postoperative full-length standing spine digital radiograph (AP/lateral view) demonstrates successful correction of scoliosis following posterior spinal fusion with pedicle screw instrumentation. The radiograph confirms proper spinal alignment, hardware positioning, and balanced sagittal/coronal profiles.

On the third postoperative day, the child’s mental state was good, and KD was changed to oral administration, and the laboratory tests gradually recovered. Postoperative blood ketone values were measured (Fig. 3). Further postoperative management was uneventful, and the child was ambulant 5 days after surgery and discharged 13 days after reexamination of the deformity on radiographs (Fig. 4). At 3 months after operation, the X-ray films showed that the internal fixation was in place and the child returned to normal life. During this period, no neurological deficits or seizures occurred in the child.

## 3. Discussion

The perioperative management of patients with scoliosis has always been a significant challenge for medical staff due to the long operation time and the high possibility of complications.^[7,8]^ Our case was not only complicated with multiple diseases, but also had nutritional specificity (KD). Therefore, we conducted a multidisciplinary team with the departments of anesthesiology, pediatrics, neurology, endocrinology and nutrition, and formulated a perfect perioperative management plan focusing on perioperative nutrition, anesthesia, blood, psychology, and other aspects. Clinicians should anticipate intraoperative metabolic fluctuations and be prepared for the unexpected by maintaining strict ketosis and hemodynamic monitoring. Proactive measures, such as avoiding pro-convulsant anesthetics and titrating antiepileptic drug levels, are critical to minimize the risk of seizures and ensure patient safety. Because the destruction of blood ketone levels, surgery, fasting, psychological, and other factors can induce seizures, so perioperative management is very important.

**Figure 5.**
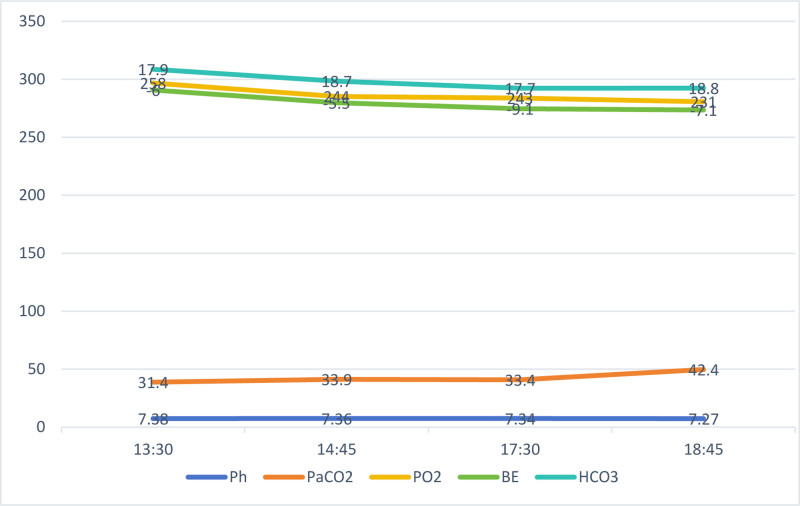
Intraoperative blood gas analysis trends over time. The bar graph depicts the standard macronutrient distribution for a ketogenic diet, expressed as a percentage of total caloric intake: 73% fat, 17% carbohydrates, and 10% protein. This ratio (approximately 4:1 fat-to-nonfat calories) is designed to induce sustained ketosis by minimizing carbohydrate availability and prioritizing lipid metabolism. The profile aligns with classical KD protocols for neurological conditions such as refractory epilepsy. KD = ketogenic diet.

Therefore, during the perioperative period, we continued to use KD and reduced carbohydrate intake to maintain blood ketone levels, thereby reducing the risk of seizures.^[9,10]^ It has been reported that the use of saline and lactated Ringer’s solution may be relatively safe, and possible complications and management have been mentioned.^[11–14]^ We performed fluid management (Table 2) and blood gas analysis (Fig. 5) as described above. The results showed no obvious abnormalities.

**Table 2:** Intraoperative volume (mL).

Intraoperative intake		
Peripheral vein	Compound sodium chloride	500 mL
	Cefazolin sodium	100 mL
	Tranexamic acid	100 mL
	Compound sodium chloride	500 mL
Central vein	Lactated Ringer’s solution	500 mL
	Autologous blood	300 mL
	Compound sodium chloride	500 mL
Total	2500 mL	

Oral KD at 6 am on the day of surgery can not only avoid the risk of hypoglycemia increased by prolonged fasting, but also meet the requirements of preoperative fasting. Jan Hudec et al^[6]^ also reported the safety and efficacy of administering KD before surgery. On the second day after surgery, the patient did not exhaust and defecate, and intravenous KD was continued while promoting defecation. On the third day after operation, oral KD was given instead of intravenous KD after defecation. Although some studies have found that a series of complications may occur with prolonged intravenous KD, excessive intravenous KD for a short period of time is safe,^[15]^ and no venous catheter-related complications occurred during this period.

The blood ketone level was within the normal range (0.03–0.5 mmol/L) during most of the perioperative period. Only on the morning of the second postoperative day, the blood ketone level was above the normal range (5.1 mmol/L) and gradually decreased to the normal range after intravenous infusion of KD. We analyzed that this may be due to the fact that in the starvation state, the body does not have enough glycogen to supply energy and the liver produces ketone bodies for the body to use, because the blood ketone bodies gradually decreased to normal levels after intravenous injection of KD. During this process, the child did not develop discomfort symptoms and did not induce seizures, which we considered to be acceptable.

The choice of anesthetic to KD children equally crucial, always think of propofol represents the total intravenous anesthesia and IONM the “gold standard” of narcotic drugs, is widely used in the spine and neurosurgery. However, propofol contains a large amount of carbohydrates, which may induce or aggravate the symptoms of epilepsy in children. Several studies reported that the latest remimazolam can replace propofol is ideal anesthetic, and can realize high quality IONM as.^[6,16,17]^ Therefore, we applied the fentanyl (analgesic), benzene sulfonic acid remimazolam maintain anesthesia (calm), IONM was performed.

Standard perioperative management for scoliosis should encompass preoperative, intraoperative, and postoperative care. Key components include respiratory function training, intraoperative wake-up test preparation, vital sign monitoring, pain management, as well as complication observation and nursing. In this particular case, the patient requires enhanced parental involvement during the perioperative period—especially necessitating additional training for intraoperative wake-up protocols or potential direct parental participation during surgery. This underscores the critical importance of training the patient’s mother to achieve proficiency in gowning and sterile techniques. Additionally, frequent monitoring of the child’s blood ketone levels is required, consistent with findings reported in the literature.^[10,12]^

In this case, the child with intellectual disability and autism was 12 years old in actual age, but only 5 to 6 years old in psychological age. Psychological management of the child was particularly important. We used simple language and physical expression to communicate with the patient, and gained his trust. And training children mother wear brush off hand clothes, into the operating room rules and regulations and aseptic principles, before the start of the anesthesia by children with mother to the escort, eliminate children with tension. At the end of the operation, the patient developed irritability, crying, and poor cooperation. Under the comfort of the mother and the effect of sedative drugs, the child’s mood gradually stabilized.

Although many studies have demonstrated the effectiveness of KD in reducing seizure frequency, significant challenges remain. Studies have shown that circulating ketones increase the acidic environment in the body, increase the risk of metabolic acidosis, and may contribute to kidney stones, nephrocalcinosis, and bone mineral density loss.^[18]^ In spinal deformity correction surgery, decreased bone mineral density can significantly increase the complexity of surgery. In addition, the choice of anesthetic agent poses a serious challenge for perioperative patients with metabolic disorders, as evidenced by the avoidance of conventional agents in this case.^[10]^ Anesthesiologists must carefully evaluate the carbohydrate content of medications, which can be a labor-intensive process. Consistent with existing literature.^[19,20]^ Parents often experience higher levels of stress and psychological burden and face great challenges in maintaining pediatric dietary adherence. The patient’s co-morbid autism spectrum disorder requires a significant investment of parental time and effort, which further exacerbates the strain of caregivers when dealing with critical illness. KD is usually administered by enteral feeding, and parenteral administration during the perioperative period has been documented.^[21]^ To maintain ketosis during KD parenteral nutrition, the daily carbohydrate intake of previous oral KD regimens should be used as the reference standard for determining the maximum carbohydrate supply of parenteral nutrition preparations. The protocol calls for vigilant monitoring to prevent KD parenteral nutrition-associated adverse effects (e.g., hypoglycemia, constipation, hyperglycemia, elevated liver enzymes, and elevated pancreatic amylase).

There were no major complications in this case, which highlights the successful implementation of multidisciplinary perioperative management. The application of KD is increasing year by year.^[22]^ This case can provide reference for the perioperative management of KD patients.

## 4. Conclusion

This case report demonstrates the feasibility of a multidisciplinary perioperative protocol for scoliosis surgery in a pediatric patient with epilepsy maintained on a KD. Key outcomes included: throughout the perioperative period, no perioperative seizures were achieved by close monitoring of ketosis and strict adherence to the KD formula. The KD metabolic interaction was adjusted by a customized anesthetic protocol. These findings emphasize that perioperative management of KD patients requires not only nutritional and analgesic precision. While this single-case study provides actionable insights, larger prospective cohorts are needed to validate standardized protocols. Future research should investigate long-term outcomes in KD patients undergoing major surgeries.

## Acknowledgments

The authors express their gratitude to the patient who made this work possible, as well as the professionals and researchers who participated in this study.

## Author contributions

**Conceptualization:** Fangjun Yang.

**Data curation:** Fangjun Yang, Dong Hou.

**Methodology:** Jiantao Wen.

**Software:** Fangjun Yang.

**Supervision:** Fangjun Yang, Jiantao Wen.

**Validation:** Fangjun Yang.

**Visualization:** Fangjun Yang.

**Writing – original draft:** Fangjun Yang.

**Writing – review & editing:** Dong Hou, Jiantao Wen.
